# Effect of add-on therapy with leukotriene receptor antagonists and Chungsangboha-tang in patients with asthma: a protocol for a randomized, placebo-controlled, parallel, multicenter trial

**DOI:** 10.1186/s12906-025-04799-w

**Published:** 2025-02-14

**Authors:** Sung-Woo Kang, Hae-Seong Nam, Yang-Chun Park, Jun-Yong Choi, Ki-Tae Kim, Seo-Jung Ha, Kwan-Il Kim, Hee-Jae Jung, Beom-Joon Lee

**Affiliations:** 1 Department of Clinical Korean Medicine, College of Korean Medicine, Graduate School, Kyung Hee University, Seoul, 02447 Republic of Korea; 2 Division of Pulmonology, Department of Internal Medicine, Inha University Hospital, Inha University School of Medicine, Incheon, 22332 Republic of Korea; 3 Department of Internal Medicine, College of Korean Medicine, Daejeon University, Daejeon, 35235 Republic of Korea; 4 Department of Internal Medicine, School of Korean Medicine, Pusan National University, Yangsan, 50612 Republic of Korea; 5 Department of Korean Medicine, College of Korean Medicine, Semyung University, Jecheon, 27136 Republic of Korea; 6 Department of Acupuncture and Moxibustion Medicine, College of Korean Medicine, Sangji University, Wonju, 26339 Republic of Korea; 7 Division of Allergy, Immune and Respiratory System, Department of Internal Medicine, College of Korean Medicine, Kyung Hee University, Kyung Hee University Medical Center, Seoul, 02447 Republic of Korea

**Keywords:** Asthma, Chungsangboha-tang, Herbal medicine, Korea traditional medicine, Leukotriene receptor antagonists

## Abstract

**Background:**

Asthma is a chronic disease characterized by airway inflammation and obstruction. Treatment aims to control symptoms with minimal medication, using disease-controlling and symptom-relieving drugs. Inhaled steroids and beta2 agonists are common treatments; however, their long-term use can cause side effects. Leukotriene receptor antagonists (LTRAs) are used in combination with inhaled steroids to manage asthma because of their anti-inflammatory and bronchodilatory effects. Combining LTRAs with Chungsangboha-tang (CSBHT), a Korean medicine, may enhance their efficacy. This study aimed to evaluate the potential of CSBHT as an adjunctive therapy for asthma management in a randomized, placebo-controlled, double-blind, multicenter clinical trial.

**Methods:**

This randomized, placebo-controlled, double-blind, parallel-group, multicenter study aims to evaluate the efficacy and safety of CSBHT as an additional treatment for patients with asthma, particularly for those with LTRAs. Overall, 198 participants will be randomly divided into intervention and control groups, with the former receiving CSBHT thrice daily and the latter receiving a placebo. Follow-ups at weeks 0, 4, and 8 will include outcome measurements, medication dispensation, and adverse reaction monitoring. The primary outcome is the mean change in forced expiratory volume in one-second scores, with secondary outcomes including changes in peak expiratory flow, forced vital capacity, forced expiratory flow 25–75%, fractional exhaled nitric oxide, Asthma Control Test, Asthma Quality of Life Questionnaire, serum IgE, eosinophil count, C-reactive protein, rescue medication usage, and a descriptive analysis of the questionnaire on asthma symptoms in Korean medicine. Safety assessments will be conducted using laboratory tests, vital signs, and monitoring of adverse events. Economic evaluations will be conducted using either cost-minimization analysis or cost-utility analysis.

**Discussion:**

This trial will evaluate the efficacy, safety, and cost-effectiveness of CSBHT as an add-on therapy to LTRAs to establish its potential as an adjuvant therapy in asthma management.

**Trial registration:**

This study was registered in the Clinical Research Information Service of Korea (KCT0006005), on March 16, 2021.

## Introduction

Asthma is a persistent and challenging chronic inflammatory disease of the airways, characterized by the hypersecretion of airway mucus, airway obstruction, eosinophilic airway inflammation, and airway hyperresponsiveness [1]. While it remains an intractable condition without a cure, the primary goal of asthma management is to achieve optimal control with minimal risk [2]. Therapeutic drugs for asthma can be categorized into two main classes: controller medications, which are employed to mitigate symptoms through their anti-inflammatory effects; and reliever medications, which are reserved for symptom alleviation during acute episodes by rapidly dilating the airways [2].

Leukotriene receptor antagonists (LTRAs), which include montelukast, pranlukast, and zafirlukast, are utilized in conjunction with inhaled steroids to manage asthma due to their anti-inflammatory effects, bronchodilatory properties, ability to reduce cough, improve lung function, alleviate airway inflammation, and decrease asthma exacerbations [3]. While LTRAs are less effective than inhaled corticosteroids (ICSs) [4], the 2024 GINA guideline suggests that they can be adopted as a maintenance method as an alternative to increasing ICS dosage [2]. The addition of LTRAs in patients already on ICS or ICS-long-acting beta2 agonist (LABA) is beneficial in controlling asthma, preventing exacerbations, and improving lung function; however, the benefits are limited and not applicable in all populations [5–7]. Consequently, there is a growing interest in the use of supplementary medications for asthma management.

Chungsangboha-tang (CSBHT) is a traditional medicine composed of 18 different drugs that have been widely used to treat respiratory diseases in East Asian countries. In vitro, animal, and human studies conducted in Korea have reported that CSBHT is clinically effective in treating asthma because of its anti-inflammatory and immunomodulatory actions [8]. Therefore, it could be a beneficial therapeutic option, complementing standard asthma treatments with its unique efficacy.

Here, we suggest that combining LTRAs with CSBHT may enhance the effectiveness of LTRAs in controlling asthma. In this study, we aim to establish the potential of CSBHT as an adjunctive therapy for asthma management by evaluating its efficacy, safety, and cost-effectiveness as an add-on therapy to LTRA in a randomized, placebo-controlled, double-blind, parallel-group, multicenter clinical trial. This study used a placebo that corresponds 1:1 to CSBHT as a control, as the placebo is the most stringent method for testing therapeutic efficacy and it is a method that can evaluate the real additional efficacy for add-on treatments to standard therapy [9]. With this study, we hope to present a standardized treatment approach that may pave the way for future health insurance coverage.

## Methods and analysis

### Trial design

This clinical trial is a randomized, placebo-controlled, double-blind, parallel-group, multicenter study that aims to ascertain the efficacy and safety of applying a treatment method that adds CSBHT to patients with asthma, a common refractory airway disease, who have received standard treatment at each stage, particularly those administered LTRAs. The study plans to recruit participants from the Department of Pulmonology and Allergy of Kyung Hee University Hospital and Inha University Hospital in South Korea, targeting patients with asthma receiving standard treatment. The clinical trial will be conducted at the Clinical Trial Centers of Kyung Hee University Korean Medicine Hospital, Inha University, Pusan National University Korean Medicine Hospital, Daejeon University Korean Medicine Hospital, and Semyung University Korean Medicine Hospital. After explaining the clinical trial to the participants, screening will be conducted on participants for whom consent has been obtained. A total of 198 participants who meet the inclusion and exclusion criteria will be recruited as research subjects and will then be randomly assigned to the intervention and control groups in a 1:1 ratio. The intervention group will take one packet of CSBHT thrice a day, and the placebo group will take one packet of CSBHT placebo thrice a day. Participants will visit after the screening visit at 0, 4, and 8 weeks, where they will receive their medication in two separate dispensations, one at 0 weeks and the other at 4 weeks. At each visit, evaluations for this study will take place, which will encompass monitoring for adverse reactions. This research protocol complies with the Good Clinical Practice guidelines, the Helsinki Declaration, and the Standard Protocol Items: Recommendations for Interventional Trials (SPIRIT) statement. This clinical trial has been registered with the Clinical Research Information Service (https://cris.nih.go.kr/cris/en/) under registration number KCT0006005.

**Fig. 1:**
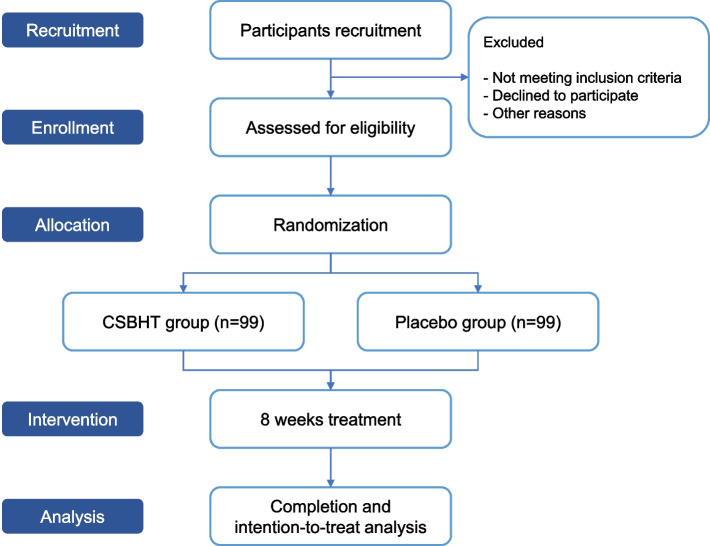
Study flow chart

Figure 1 presents the research flow, while Table 1 outlines the research schedule.

**Table 1:**
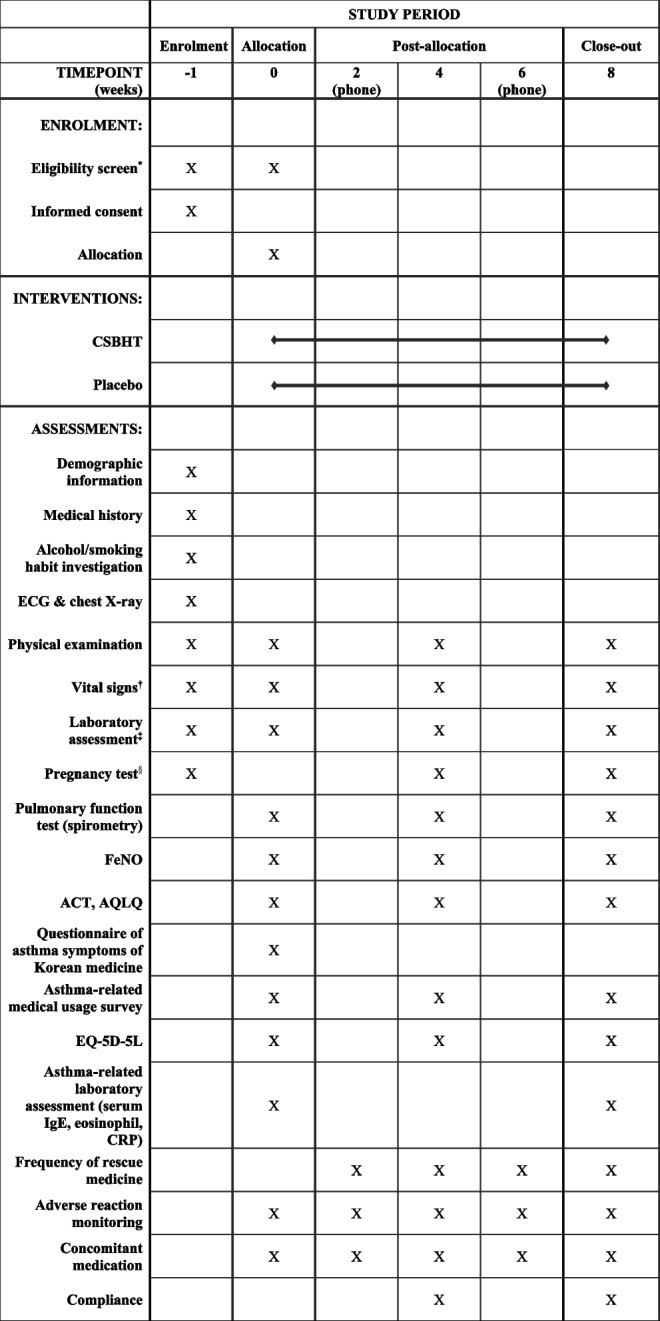
Timeline for the study protocol

*CSBHT* Chungsangboha-tang, *ECG* electrocardiogram, *FeNO* fractional exhaled nitric oxide, *ACT* Asthma Control Test, *AQLQ* Asthma Quality of Life Questionnaire, *EQ-5D* EuroQol 5-dimension, *CRP* c-reactive protein, *AST* aspartate aminotransferase, *ALT* alanine aminotransferase, *ALP* alkaline phosphatase, *TB* total bilirubin, *GGT* gamma glutamyl transferase, *BUN* blood urea nitrogen, *Na* sodium, *K* potassium, *Cl* chloride, *WBC* white blood cell count, *RBC* red blood cell count, *eGFR* estimated glomerular filtration rate

^*^Screening tests should be conducted within 7 days prior to Visit 1 (0 weeks), and laboratory assessments including AST, ALT, ALP, TB, GGT, BUN, creatinine, Na, K, Cl, WBC, RBC, hemoglobin, hematocrit, and platelet count should be confirmable on Visit 1. For suitable candidates, Screening and Visit 1 can be conducted simultaneously

^†^Vital signs include measurements of blood pressure, pulse, and body temperature

^‡^Laboratory assessments include the following:

• Blood chemistry tests: AST, ALT, ALP, TB, GGT, BUN, creatinine, eGFR, Na, K, Cl

• Hematology tests: WBC. RBC. hemoglobin, hematocrit, platelet count

^§^A human chorionic gonadotropin test conducted via urinalysis (only for women with the possibility of pregnancy)

### Participants

#### Inclusion criteria

1. Men and women aged 19–85
2. Patients who have been diagnosed with asthma and treated for over 4 weeks
3. Patients who are being administered ICS-LABA and LTRA or ICS and LTRA
4. Patients who voluntarily decide to participate in this clinical trial and sign a consent form

#### Exclusion criteria

1. Patients with co-morbidities such as pneumonia, interstitial lung disease, and active pulmonary tuberculosis on chest X-ray examination
2. Patients who have been using maintenance therapy with oral steroids, anti-IgE antibodies, or herbal medicines for asthma treatment since 4 weeks ago.
3. Patients with aspartate aminotransferase (AST)/alanine aminotransferase (ALT) levels exceeding twice the normal limit, or with an estimated glomerular filtration rate (eGFR) of less than 60 mL/min/1.73 m^2^ (However, for those aged 65 and over, individuals with an eGFR less than 45 mL/min/1.73m^2^)
4. Chronic heart failure of class III or IV (New York Heart Association)
5. Patients with alcohol or other substance abuse/dependency or history
6. Patients with malignant tumors or a history of malignant tumors (however, if they have not recurred for more than 5 years, participation is possible).
7. Patients with a history of hypersensitivity reactions or allergies to research-related drugs
8. Patients who are unable to read and write or have cognitive impairment
9. Pregnant or lactating women
10.  Patients who participated in other clinical trials within recent 30 days before this clinical trial (based on the date of written consent)
11.  If the researcher determines that it is inappropriate to participate in clinical research due to other reasons

#### Withdrawal criteria

1. Cases where the participant received treatments that could influence the study outcomes without the researcher's instructions during the study period
2. Cases where the participant did not adhere to the clinical trial protocol or the compliance was below 70%
3. Cases where the participant experienced adverse reactions that made it difficult to continue the clinical trial or where severe adverse reactions occurred
4. Cases where the participant withdrew their consent
5. Cases where the participant used additional drugs or any herbal medicine that could affect asthma treatment during the clinical trial (excluding temporary short-term use for treating acute exacerbation of asthma)
6. Cases where the LTRA prescription was changed after the participant's registration
7. Other reasons deemed by the researcher as inappropriate for the continuation of the clinical trial

### Randomization, allocation concealment, and blinding

Study participants will be randomly assigned in a 1:1 ratio to the intervention and control groups. Random assignment is performed by an independent statistician using SAS V.9.1.3 for Microsoft Windows (SAS Institute Inc., NC, Cary, USA) to generate random numbers and construct a random assignment table. The random number table and the intervention or placebo group assignment table created using SAS will be retained by a third party until the termination or completion of the study, with no access granted to the researchers. Written consent to participate in the study will be obtained by the principal investigator or sub-investigators, who are doctors and Korean medicine doctors authorized by the principal investigator. Once the study participants are informed about the study and have signed a consent form for participation in the clinical trial, they are assigned an institution number for each site and a screening number. After screening, suitable participants are assigned random numbers. The study participants who are finally selected to participate in the clinical trial through the screening test are given a registration number. Based on the random assignment table held by a third party, they are assigned to a group corresponding to their registration number. The manufacturers of the investigational drugs and placebos directly conduct code labeling assigned to the investigational drugs and placebos, and a third party matches the generated random numbers and codes. The investigational drugs and placebos will be stored in the clinical trial pharmacy of each institution and delivered to the clinical participants by a managing pharmacist who maintains blinding.

The investigational drug and placebo are managed using assigned codes, implementing a double-blind method, in which the researchers, outcome assessors, data analysts, and trial participants are unaware of the type of drug being administered. During the clinical trial period, if the blinding must be lifted before the completion of the clinical trial due to a serious adverse reaction, the research participant contacts a third party and proceeds with the blinding release procedure after reviewing and approving the reason for the blinding release.

### Sample size

The current study requires a total of 168 test subjects. Considering a dropout rate of 15%, the sample size will be adjusted to 198.

The required number of recruits per group was calculated with reference to the study conducted in 2019 [10]. According to the reference literature, the changes in forced expiratory volume in the first second (FEV_1_) after 8 weeks of Bufei granule administration in a total of 178 subjects, with 89 in the test group and 89 in the control group, are as follows (Table 2).

**Table 2:** Changes in FEV_1_ (L) observed in the study by Xin X and Peng F (2019) [10]

	FEV_1_ (L) before treatment	FEV_1_ (L) after treatment
Test group (*n* = 89)	0.88 ± 0.30	1.72 ± 0.75
Control group (*n* = 89)	0.89 ± 0.25	1.37 ± 0.80

As the primary outcome of this study is the variation in FEV_1_, the calculation of FEV_1_ changes was based on the aforementioned study. The mean change in FEV_1_ in each group was calculated as the difference between the post-treatment and pre-treatment means. The standard deviation was calculated based on the following formula, considering the pre-treatment standard deviation, post-treatment standard deviation, and the correlation between measurements taken pre- and post-treatment:

$$\mathrm{Mean}\;\mathrm{change}\;\mathrm{in}\;{\mathrm{FEV}}_1\;=\;\mathrm{Post}-\;\mathrm{treatment}\;\mathrm{mean}\;\;-\;\;\mathrm{Pre}-\mathrm{treatment}\;\mathrm{mean}$$

$$\mathrm{Standard}\;\mathrm{deviation}\;\mathrm{of}\;{\mathrm{FEV}}_1\;\mathrm{change}\;=\;{({\mathrm{Pre}-\mathrm{treatment}\;\mathrm{standard}\;\mathrm{deviation}}^2\;+\;{\mathrm{Post}-\mathrm{treatment}\;\mathrm{standard}\;\mathrm{deviation}}^2\;-\;2\;\ast\;\mathrm{Correlation}\;\mathrm{coefficient}\;\mathrm{between}\;\mathrm{pre}-\;\mathrm{and}\;\mathrm{post}-\mathrm{treatment}\;\mathrm{measurements}\;\ast\;\mathrm{pre}-\mathrm{treatment}\;\mathrm{standard}\;\mathrm{deviation}\;\ast\;\mathrm{post}-\mathrm{treatment}\;\mathrm{standard}\;\mathrm{deviation})}^{(1/2)}$$

The correlation coefficient between the pre- and post-treatment measurements was conservatively assumed to be zero because of a lack of related references. The calculated FEV_1_ change from this reference paper was found to be 0.39 ± 0.838 for the control group and 0.84 ± 0.808 for the test group.

In this study, the primary outcome is the change in FEV_1_, with a significance level (α) set at 0.05 and a test power (1-β) set at 0.8. The number of participants was determined using g-power, considering a two-sided test and an allocation ratio of 1:1 [11]. As a result, the effect size was calculated to be 0.437, and the total number of participants was 168. Taking into account the number of recruitable participants, the minimum scope for validity assessment, and a dropout rate of 15%, the total number of participants was determined to be 198 to secure the minimum necessary sample size.

### Interventions

#### Drug interventions

The CSBHT and placebo were designed with the same weight (1.8 g/pack) and packaging. CSBHT is composed of the following main components: Rehmanniae Radix Preparata (187.5 mg), Dioscoreae Rhizoma (93.5 mg), Corni Fructus (93.5 mg), Poria Sclerotium (70.0 mg), Moutan Radicis Cortex (70.0 mg), Alismatis Rhizoma (70.0 mg), Schisandrae Fructus (70.0 mg), Ponciri Fructus Immaturus (70.0 mg), Liriopis seu Ophiopogonis Tuber (70.0 mg), Asparagi Tuber (70.0 mg), Fritillariae Thunbergii Bulbus (70.0 mg), Platycodonis Radix (70.0 mg), Coptidis Rhizoma (70.0 mg), Armeniacae Semen (70.0 mg), Pinelliae Tuber (70.0 mg), Trichosanthis Semen (70.0 mg), Scutellariae Radix (70.0 mg), and Glycyrrhizae Radix et Rhizoma (23.25 mg). The placebo is similar to CSBHT in appearance, taste, and smell, but does not contain any active ingredients. It comprises lactose (252.0 mg), corn starch (1030.0 mg), PEG6000 (233.0 mg), gum arabic (140.0 mg), HPC-M (80.0 mg), CMC-na (40.0 mg), yellow iron oxide (0.4 mg), black iron oxide (0.4 mg), red iron oxide (0.2 mg), caramel color (12 mg), and Opadry Brown amb2 (12 mg). Both CSBHT and the placebo are produced by Hanpung Pharmaceuticals, according to the pill section of the General Rules of Formulation in the Korean Pharmacopoeia. The intervention and control groups will take either the herbal compound CSBHT or the CSBHT placebo, both of which are in pill form, at a dose of 1.8 g/pack, three times a day for a total of eight weeks. They will visit 0, 4, and 8 weeks after the screening visit, receiving a total of two prescriptions at 0 and 4 weeks, respectively. At each visit, evaluations included in this study, including any adverse reactions, will be conducted. Compliance with medication is defined as the amount of clinical trial medication completed during the clinical trial period divided by the total amount to be taken; and if this value is < 70%, they will be dropped from the study.

$$Compliance\mathit\;(\%)\mathit=\mathit\;\frac{\mathit D\mathit o\mathit s\mathit a\mathit g\mathit e\mathit\;\mathit a\mathit d\mathit m\mathit i\mathit n\mathit i\mathit s\mathit t\mathit e\mathit r\mathit e\mathit d}{\mathit D\mathit o\mathit s\mathit a\mathit g\mathit e\mathit\;\mathit p\mathit l\mathit a\mathit n\mathit n\mathit e\mathit d}\mathit\;\times\;100$$

$$Dosage\;administered=Dosage\;prescribed\;-\;Dosage\;returned$$

### Concomitant medications

During the clinical trial participation period, the participant is advised not to take any medications other than the investigational product. However, if another medication is administered in combination at the investigator’s discretion, information regarding the medication (such as name, daily dose, administration period, and reason for administration) must be recorded in the case report form (CRF). Drug history is checked for all preceding/concurrent medications within four weeks based on the screening point.

The use of new medications that influence the results of a clinical trial during the administration period of an investigational product is prohibited. The use of emergency medications is not prohibited if a patient's asthma-related symptoms worsen temporarily. However, if a patient's asthma symptoms persistently worsen and additional medications such as systemic oral steroids, theophylline, other xanthine drugs, anti-IgE antibodies, and persistent anticholinergic bronchodilators are needed for continuous control, the clinical trial is halted and the relevant medication is administered. This is not applicable in cases where they are used momentarily for the short-term treatment of acute exacerbation of asthma.

### Outcome measurements

#### Primary outcome

##### Pulmonary function test (Forced Expiratory Volume in 1 s, FEV_1_)

Spirometry is a test that plays an essential role in the diagnosis and monitoring of asthma to identify airflow limitation [2]. Using spirometry, measurements of forced vital capacity (FVC), FEV_1_, among others are taken, and changes in FEV_1_% and liters are observed to measure the amount of change. The primary outcome of this study will be the average change in FEV_1_ scores between baseline and week 8.

#### Secondary outcomes

##### Pulmonary function tests (Peak Expiratory Flow, PEF; Forced Vital Capacity, FVC; Forced Expiratory Flow at 25–75%, FEF25–75)

Among the items tested by spirometry, PEF, FVC, and FEF25–75 are measured, and changes in % and liters are observed to measure the amount of change. PEF, FVC, and FEF25–75 will be evaluated at baseline and weeks 4 and 8.

##### Fractional exhaled nitric oxide (FeNO)

Fractional exhaled nitric oxide (FeNO) is a test utilized to ascertain the severity of bronchial inflammation, by measuring a substance known as nitric oxide present in the exhaled air [12]. The measurement is conducted by having the subject exhale at a constant rate for ~ 10 s into a mouthpiece connected to a monitor, thereby determining the concentration of nitric oxide. FeNO scores will be evaluated at baseline and weeks 4 and 8.

##### Asthma Control Test (ACT)

The Asthma Control Test (ACT) comprises five questions related to the frequency of asthma symptoms and the use of rescue medication over the past four weeks. Scores are interpreted as follows: 15 or below indicates a very poorly controlled condition, 16 to 19 indicates a poorly controlled condition, and 20 to 25 signifies a well-controlled condition [2]. The ACT will be evaluated at baseline and weeks 4 and 8.

##### Asthma Quality of Life Questionnaire (AQLQ)

The Asthma Quality of Life Questionnaire, AQLQ is a 32-item questionnaire used to assess the physical, occupational, emotional, and social characteristics of adults aged 17–70 with asthma [13]. The AQLQ is fully validated and effective in evaluating patients exhibiting mild to moderate asthma [14]. The AQLQ is composed of four domains: symptoms (12 items), activity limitations (6 general items and 5 patient-specific items), emotional function (5 items), and environmental stimuli (4 items). The AQLQ will be evaluated at baseline and weeks 4 and 8.

##### Questionnaire of asthma symptoms of Korean medicine 

The questionnaire on asthma symptoms in Korean medicine is evaluated at baseline and presented in a descriptive analysis.

**Deficiency and excess pattern identification questionnaire**

 The tool for assessing the deficiency and excess pattern identification of asthma patients used the questionnaire listed in the “Guideline for clinical trials with herbal medicinal products: antiasthmatic agents” presented by the Korea Food & Drug Administration [15]. This questionnaire was developed by modifying the deficiency and excess pattern identification items used in previous research, and the patient's asthma pattern is largely divided into deficiency and excess.

Excess patterns are divided into exogenous wind cold and excessive internal phlegm dampness, the latter subdividing into phlegm dampness, cold phlegm, and phlegm heat, and deficiency patterns are divided into lung deficiency, heart-kidney deficiency, and upper excess with lower deficiency, classifying asthma into seven pattern identifications. The total score for each pattern identification unit is 10 points for excess pattern and 11 points for deficiency pattern; only those with 5 points or more are recognized as pattern identifications. If there are two or more pattern identifications with five or more points, the one with the highest score is selected as the pattern identification. If the scores are the same, the pattern identification with greater weight is selected and classified.

**Cold-heat pattern questionnaire**

The asthma cold-heat pattern model of the patient used the Cold-Heat Pattern Questionnaire presented by Ryu et al. (2008) [16]. This questionnaire consists of 8 items on cold patterns and 7 items on heat patterns in six categories such as ‘thirst,’ ‘restlessness,’ ‘constipation,’ ‘chills,’ ‘cold hands and feet,’ and ‘diarrhea.’ The responses are measured using a 7-point Likert scale, which ranged from 'Not at all,’ ‘Not really,’ ‘Somewhat no,’ 'Neutral,’ 'Somewhat yes,’ ‘Yes,’ to 'Absolutely,’ based on the frequency and severity of the symptoms. The heat and cold scores are calculated as the sum of the averages of the weight categories. The heat and cold indices are calculated as percentages of the occupancy of the cold-heat attribute by dividing the heat and cold scores by the maximum heat and cold scores, respectively.

##### Serum IgE, eosinophil count, c-reactive protein (CRP)

Asthma is a disease characterized by chronic inflammation of the airways and is associated with elevated levels of blood eosinophils, immunoglobulin E (IgE), and inflammation-related indices such as CRP [17]. We plan to quantify these changes and assess their correlation with the disease progression. Serum IgE, eosinophil count, and CRP will be measured at baseline and weeks 4 and 8.

##### Frequency of rescue medicine

According to the Korean Asthma Management Guidelines (2015) [18], when symptoms of caution-level acute exacerbation occur, as presented in the asthma action plan during asthma patient management education, the emergency drug Ventolin Evohaler from GlaxoSmithKline is used, and its usage frequency is measured. The criteria presented at the caution level are as follows: 1. One or more symptoms, such as cough, wheezing, chest tightness, or shortness of breath are present. 2. Waking up at night because of asthma symptoms. 3. Interference with daily activities. 4. The peak expiratory flow rate is between 60–80% of the individual's maximum. In this case, symptom relievers (Ventolin Evohaler) are sprayed twice each, up to three times. The number of uses of the rescue medication will be evaluated at baseline and weeks 4 and 8. Additionally, assessments will be conducted via telephone visits at weeks 2 and 6.

### Safety assessment

Laboratory tests (liver function test, blood urea nitrogen (BUN), creatinine, eGFR, sodium, potassium, and chloride) and vital sign measurements will be conducted before drug administration and 4 and 8 weeks after administration. During the 2nd and 6th weeks of drug administration, adverse reactions will be checked via phone visits to evaluate any adverse reactions associated with the drug administration during the test period. If an adverse reaction occurs, a trial participation meeting will be held to estimate its association with the clinical drug.

To test the safety of the herbal medicine intake, AST, ALT, alkaline phosphatase (ALP), total bilirubin (TB), gamma glutamyl transferase (GGT), BUN, creatinine, and eGFR are measured. To check for damage to liver function caused by herbal medicine, AST, ALT, ALP, TB, and GGT levels are measured. This indicator was structured with reference to the herbal medicine-induced liver injury investigation forms to improve the research quality of herbal safety presented by Yoon et al. (2009) [19]. The presence of kidney function damage caused by herbal medicine intake is confirmed by BUN, creatinine, and eGFR.

Adverse events (AEs) refer to undesirable and unintended signs, symptoms, or diseases that occur during a clinical trial and do not necessarily have to be causally related to the treatment used in the clinical trial. Serious adverse events (SAEs) refer to medical situations that are of significance, including death, life-threatening situations, need for hospitalization or extension of hospitalization, permanent or severe disability and impairment, and congenital anomalies or abnormalities occurring in the fetus, among the AEs that occur in the clinical trial participants during the clinical trial.

Continuous AE monitoring is conducted for all participants throughout the trial period. If the person in charge of the clinical trial determines a clinically significant change through an objective examination, subjective symptom questioning, and blood tests, it is considered an AE. The researcher records all information related to the AE and SAE in the CRF, including the AE name, occurrence date, end date, intensity, correlation with the investigational drug, outcome, treatment status, and SAE status.

If an AE deemed to harm the participant is observed at any time during the clinical trial period, the researcher can temporarily suspend treatment. When the progression of the AE is tracked, its causal relationship with the treatment is assessed, and it is determined that the continuation of the treatment could harm the subject, the researcher will permanently discontinue the patient's participation in the clinical trial. The treatment in the clinical trial can be temporarily suspended or continued at the discretion of the researcher if the existing disease worsens during the clinical trial period. The clinical trial is immediately and permanently discontinued for all pregnant subjects. If any AE occurs that necessitates medical attention as determined by the investigator, appropriate action will be taken in accordance with the rules for compensating victims through insurance coverage.

### Statistical analysis

#### Effectiveness outcomes

Statistical analyses are conducted independently by a statistical expert using SAS. In the two-tailed tests, the level of significance is set at 0.05. All analyses primarily utilize intention-to-treat analysis, with per-protocol analysis conducted concurrently, and the results are presented together. In the demographic baseline data, continuous variables are presented as mean and standard deviation and are analyzed using an independent t-test or Mann–Whitney U test. Categorical variables are presented as frequency and proportion and analyzed using a chi-square test or Fisher's exact test (when the expected frequency is less than 5 in more than 25% of all values).

The primary efficacy evaluation is based on the mean change in FEV_1_ scores evaluated at week 8 (56 days post-dosing), whereas secondary efficacy evaluations compare the means of PEF, FVC, FEF25–75, FeNO, ACT scores, AQLQ scores, Serum IgE, eosinophil count, CRP, and the frequency of rescue medication use assessed at baseline and weeks 4 and 8. If the assumption of normality is satisfied, an independent t-test is conducted; otherwise, the Mann–Whitney U test is applied. However, if significant differences between the groups are found in baseline values, an analysis of covariance (ANCOVA) is performed. Missing data is replaced using the last observation carried forward method; each missing value is replaced with the participant's last observed value. For the secondary efficacy evaluation, the distribution of participants according to each diagnosis of Korean medicine pattern identification assessed at baseline is presented as frequency and proportion and visualized in tables or graphs through descriptive analysis.

Subgroup analysis will be performed according to the following criteria: First, subgroups will be divided according to the type of asthma medication (ICS or ICS + LABA) taken in addition to the LTRA at enrollment. Second, the effect will be further analyzed by age, separating younger patients (< 35 years) from older patients (> 60 years). Third, subgroups will be defined as male and female. Fourth, subgroups will be created based on severity as measured by ACT, ≤ 15 and > 15. Finally, subgroup analyses will be performed according to Korean medicine pattern identification.

### Safety analysis

All adverse reactions that have occurred are presented by severity and causal relationship with the test drug, and the incidence rates of adverse reactions, adverse reactions that caused dropout, and serious adverse reactions are analyzed. For clinical trial subjects who dropped out due to treatment failure or adverse reactions, the proportion of each group is presented along with a 95% confidence interval. Chi-square analysis or Fisher's exact test is performed on the observed and expected frequencies.

The mean differences in AST, ALT, ALP, TB, GGT, BUN, creatinine, and eGFR levels at baseline, week 4, and week 8 are compared. If the assumption of normality is satisfied, an independent t-test is performed, and if the assumption of normality is not met, a Mann–Whitney U test is conducted. However, if a significant difference between the groups is found in the baseline values of AST, ALT, ALP, TB, GGT, BUN, creatinine, and eGFR, an ANCOVA is performed. The missing values are estimated and replaced using the stochastic regression imputation method.

### Economic evaluation

This study serves as a preliminary investigation for economic evaluation in tandem with a clinical trial, in which an additional analysis of costs is conducted based on the outcomes of the clinical trial. Depending on the results of the clinical trial, a cost-minimization analysis or cost-utility analysis is planned. The analysis period is identical to that of the clinical trial based on the results of the fourth and eighth weeks. The perspective of the analysis is based on the healthcare system.

The costs are calculated by combining the frequency of treatments and unit costs; the primary cost components comprise direct medical costs, direct non-medical costs (transportation costs and nursing costs), and indirect costs (costs of productivity loss and time costs). Cost data are obtained using clinical trial institution data and a self-developed medical use survey form (loaded in the electronic CRF), and costs that cannot be obtained from research subjects or clinical trial institution data are estimated based on other statistical indicators.

EuroQol 5-dimension (EQ-5D) was used for the economic evaluation analysis. The EQ-5D is an indicator that indirectly measures quality weights reflecting individual preferences and evaluates general health status (quality of life) through five items (mobility, self-care, usual activities, pain/discomfort, and anxiety/depression). The EQ-5D-5L, an enhancement of the EQ-5D-3L that was developed by the EuroQol Group in 1990, was developed in 2009 [20]. In this study, the Korean version of the EQ-5D-5L with proven validity is utilized. A validity study was conducted according to the standard protocol set by the EuroQol Group, and quality-adjusted life years were calculated based on the value assessment results of Kim et al. (2016), which had representative samples [21].

All trial participants participating in the clinical trial are required to self-complete the EQ-5D at baseline, week 4, and week 8. If adverse effects are observed, additional follow-up surveys should be conducted. In addition, the measurement results collected as clinical effectiveness evaluation variables in this clinical trial (measurements at baseline, week 4, and week 8) can also be included in the analysis if necessary.

If there is no difference between the groups in the clinical research results including side effects, a cost-minimization analysis is performed, and the results of all cost items surveyed (total cost) are analyzed. If there is a difference between groups in the results of the clinical research, a cost-effectiveness analysis is conducted to present the incremental cost-effectiveness ratio (ICER), and at the same time, the total cost and total utility of each group intervention is presented to confirm the overall scale. The ICER is calculated by dividing the increment in cost compared with the control group by the increment-in-effect. As only the cost and effect of the current year are considered, no discount rate is applied. If necessary, univariate sensitivity analysis is performed for uncertain variables related to cost and effect.

### Data collection, management and monitoring

Investigators receive comprehensive training on protocol adherence, processes for measuring outcomes, and data recording to ensure the validity and reliability of the data. All data will be entered into the electronic CRF under the supervision of trained investigators. To ensure data accuracy, research associates will conduct thorough verification between CRF data and source documents, resolving any discrepancies. The researcher will contact participants who do not visit within the specified time frame by phone as a reminder. Participants will receive monetary incentives at each visit to encourage participation and completion of follow-up.

A separate space is prepared to preserve the various data and records related to the conduct of clinical trials, ensuring their security. Researchers will transfer clinical trial-related records and materials to the custodian of the trial conduction institution, according to relevant regulations, for preservation for three years following the completion of the trial, except for those with a specific preservation period set by other laws. Furthermore, trial conduction institutions must establish precautions to prevent these materials from being prematurely damaged or lost due to unforeseen incidents. Upon conclusion of the research, the original data collected and acquired during the clinical trial, CRF, and other research processes will be preserved separately in accordance with the regulations dictated by the Institutional Review Board of Kyung Hee University Korean Medicine Hospital.

All documents related to the clinical trial, such as CRF, will be recorded and distinguished using subject identification codes instead of patient names. The source documents associated with this research will be stored in a separate space fitted with a locking device, or on a computer with restricted access. Access to the database is limited to one monitor and the principal investigator. The research will be performed in compliance with the relevant regulations and ethical guidelines pertaining to the protection of subjects, as stipulated by each hospital.

Monitoring is carried out in clinical trials to safeguard the rights and welfare of the subjects; ensure the accuracy, completeness, and verifiability of the reported clinical trial data by cross-referencing with source documents; and confirm the trial's compliance with the approved protocol, clinical trial management standards, and implementation rules. The Clinical Trial Centers at Kyung Hee University Korean Medicine Hospital or Inha University will assign the monitoring task to HelpTrial Inc. (Seoul, Republic of Korea), a clinical research organization. HelpTrial Inc. will conduct regular institutional visits or phone calls, free from any conflicting interests. During these visits, the monitor inspects the participants’ original records, procedural management records, and data storage. The monitor closely oversees the progression of the clinical trial and collaborates with researchers to address any issues. The timing of these visits is determined through mutual agreement between the researcher and the monitor. The researcher is also required to allow monitoring access to the subjects' original documents to verify the data recorded in the CRF, as stipulated in the clinical trial management standards and implementation rules. Findings are reported directly to the principal investigator, ensuring transparency and accountability throughout the study. No interim analysis will be performed.

## Discussion

Asthma management adheres to a stepwise approach, with escalating treatment when control is not adequately achieved. Inhaled steroids serve as the cornerstone of asthma management, supplemented by beta2 agonists in a stepwise standard treatment regimen [2]. However, escalating steroid doses over extended periods may exacerbate side effects such as osteoporosis and oral candidiasis [22]. The recommendation is to delay steroid dose increments whenever possible by employing multiple disease-modifying agents in combination, thus minimizing the need for escalating steroid usage at each stage of treatment [23].

LTRA is a drug used for asthma maintenance therapy and as a supplement to standard treatment [2]. It effectively moderates the exacerbation of asthma symptoms by complementing ICS and contributes to reducing the dosage of ICS [24]. Despite a high level of awareness of asthma guidelines among physicians, there remains a notable preference for oral medications by both doctors and patients in Korea [25]. This preference is reflected in statistics from 2017, indicating that LTRAs are the most commonly prescribed medication for 48.8% of asthma patients [26]. A recent nationwide cohort study has reported the therapeutic efficacy of combining LTRA with ICS-LABA therapy in preventing acute exacerbations of asthma across all types of combinations [27].

However, the effectiveness of the co-administration of LTRAs in asthma control has sparked significant controversy. While recommended as an adjunct to inhaled steroids by the GINA guidelines [2], its efficacy is debated, especially concerning its ability to spare steroids, a fundamental aspect of treatment [6]. Moreover, montelukast has been associated with adverse effects such as depression, agitation, sleep disturbance, and suicidal ideation, leading the FDA to recommend that it be avoided in mild asthma and allergic rhinitis, and to issue a cautionary warning, especially for children [28, 29]. Therefore, using LTRA as a maintenance therapy for asthma may not be sufficient for some patients, necessitating research on the potential effects of candidate drugs that could supplement LTRA as a new treatment.

CSBHT is a well-regarded herbal medicine used to treat respiratory conditions, attributed to its anti-inflammatory effects and the positive clinical outcome observed in numerous studies [8]. However, no randomized controlled trials have been conducted on the efficacy of CSBHT, thus failing to prevent the risk of bias. Further investigation is required because prior research on the efficacy of combining LTRA and CSBHT is lacking. Therefore, in this multicenter, randomized, double-blind, placebo-controlled trial, we plan to provide high-quality evidence on the effectiveness, safety, and cost-effectiveness of CSBHT by evaluating various outcomes of adding CSBHT as an adjuvant treatment for LTRA patients with asthma. If CSBHT is proven effective, safe, and cost-effective as an adjunctive treatment for LTRA, it will provide a basis for adding CSBHT as an adjuvant medication to the standard treatment of asthma in future medical practice.

## Data Availability

No datasets were generated or analysed during the current study.

## Abbreviations

AE: Adverse events
ALT: Alanine aminotransferase
ALP: Alkaline phosphatase
ANCOVA: Analysis of covariance
AST: Aspartate aminotransferase
ACT: Asthma Control Test
AQLQ: Asthma Quality of Life Questionnaire
BUN: Blood urea nitrogen
CRP: C-reactive protein
CRF: Case report form
CSBHT: Chungsangboha-tang
eGFR: Estimated glomerular filtration rate
EQ-5D: EuroQol 5-dimension
FEF25–75: Forced expiratory flow at 25–75%
FEV: Forced expiratory volume
FVC: Forced vital capacity
FeNO: Fractional exhaled nitric oxide
GGT: Gamma glutamyl transferase
IgE: Immunoglobulin E
ICER: Incremental cost-effectiveness ratio
ICS: Inhaled corticosteroid
LABA: Long-acting beta2 agonist
LTRA: Leukotriene receptor antagonist
PEF: Peak expiratory flow
SAE: Serious adverse event
SPIRIT: Standard Protocol Items: Recommendations for Interventional Trials
TB: Total bilirubin
